# Inhibition of Low-Density Lipoprotein Oxidation by Cysteamine, Cystamine, Cysteine and Cystine at Lysosomal pH and pH 7.4

**DOI:** 10.3390/antiox15010020

**Published:** 2025-12-22

**Authors:** Emily J. Parkes, Ana M. Cruz, Amanpreet Kaur, Georgina R. Clark, Thomas E. Pulford, Christopher Ness, Feroz Ahmad, Yichuan Wen, David S. Leake

**Affiliations:** School of Biological Sciences and Institute for Cardiovascular and Metabolic Research, University of Reading, Reading RG6 6UR, UK; e.j.parkes@pgr.reading.ac.uk (E.J.P.); anamiguelcruz@live.co.uk (A.M.C.); amanpreet.kaur@reading.ac.uk (A.K.); thomas.pulford@live.co.uk (T.E.P.); christopherness.zoso@gmail.com (C.N.); ferozahmad85@gmail.com (F.A.)

**Keywords:** atherosclerosis, LDL oxidation, foam cells, lysosomes, cysteamine, cystamine

## Abstract

LDL can be oxidised in the lysosomes of macrophages. Cysteamine, a thiol antioxidant that accumulates in lysosomes, inhibits the oxidation of LDL by iron at lysosomal pH (pH 4.5) and protects against atherosclerosis in mice. We have investigated the effects of cysteamine and its related thiol cysteine and their disulfides on LDL oxidation by iron or copper at both pH 4.5 and 7.4. The oxidation of LDL by ferrous iron (5 µM) at pH 4.5 was delayed 12.9-fold by 100 µM cysteamine and 5.6-fold by 100 µM cysteine. Cystamine and cystine (the disulfide oxidation products of cyteamine and cysteine, respectively) did not inhibit LDL oxidation by ferrous iron at pH 4.5. LDL oxidation by 5 µM copper at pH 4.5 was delayed about 2-fold by 100 µM of the thiols cysteamine and cysteine, but there was little effect of the disulfides cystamine and cystine. Cysteamine and cystine did not inhibit the oxidation of LDL by ferrous iron at pH 7.4 in a MOPS buffer and even accelerated LDL oxidation later in the incubation. Cysteine initially inhibited the oxidation of LDL by ferrous iron at pH 7.4, but increased it later. LDL oxidation by copper at pH 7.4 was delayed 7.8-fold by 100 µM cysteamine. Cysteine delayed LDL oxidation by copper at pH 7.4 to a similar extent as cysteamine but, unlike cysteamine, continued to decrease the rate of oxidation even after the period of total inhibition had ended. Cystamine had no effect on LDL oxidation by copper at pH 7.4, but cystine partially inhibited LDL oxidation. The effects of thiols and disulfides on LDL oxidation, therefore, depend not only on the metal ion catalysing the oxidation but also on the pH of the environment.

## 1. Introduction

The oxidation of low-density lipoprotein (LDL) is widely believed to be important in the pathogenesis of atherosclerosis [1,2,3]. The oxidised LDL hypothesis proposes that cells in the arterial wall oxidise LDL in the extracellular space and then endocytose it rapidly, leading to the formation of foam cells [4]. Two problems with the conventional oxidised LDL hypothesis are that the oxidation is inhibited by low concentrations of interstitial fluid [5] and that large clinical trials have shown no protection by the antioxidants vitamins E and C against cardiovascular disease [6,7,8,9,10]. We have therefore hypothesised that LDL aggregated by enzymes, such as sphingomyelinase, secretory phospholipase A_2_s or proteases, in the extracellular space of atherosclerotic lesions is phagocytosed by macrophages, delivered to lysosomes and oxidised within these organelles [11]. After taking up LDL aggregated by vortexing or by incubation with sphingomyelinase mouse or human macrophages generated the advanced lipid oxidation product ceroid [12] in their lysosomes [11,13]. Furthermore, 7-ketocholesterol, one of the main products of LDL oxidation [14], was generated in macrophages after they were treated with acetylated LDL, which is also rapidly endocytosed [11]. Chloroquine, which increases the pH of lysosomes, inhibited the oxidation of LDL [11]. This might explain how LDL can be oxidised in atherosclerotic lesions, despite the presence of numerous antioxidants in interstitial fluid [5].

Others have demonstrated that cholesterol-laden foam cells in human atherosclerotic lesions contain catalytically active iron in their lysosomes [15,16,17,18]. Iron can oxidise LDL effectively at the lysosomal pH of 4.5, as measured by spectrophotometry [11], and the iron chelator desferrioxamine inhibits the lysosomal oxidation of LDL in macrophages [11]. Lysosomes also contain copper [19] and this might contribute to LDL oxidation in these organelles.

Cysteamine (2-aminoethanethiol) is already used in patients for the rare lysosomal storage disease cystinosis, caused by the absence of the lysosomal cystine transporter cystinosin [20]. Cysteamine reacts with the accumulated disulfide cystine inside lysosomes to form cysteine and the mixed disulfide cysteine-cysteamine, both of which can exit the lysosome by alternative transporters [21,22]. Cysteamine is administered orally from childhood and taken indefinitely, extending life expectancy from about 10 years to as much as 40 years [20].

Cysteamine is a thiol antioxidant that accumulates to high concentrations in lysosomes due to proton trapping because of its amine group. Cysteamine inhibits the formation of conjugated dienes when LDL is oxidised by iron at pH 4.5 [13]. It decreases the lysosomal oxidation of LDL in human macrophages, measured by the generation of ceroid [13], and decreases the formation of atherosclerosis in LDL receptor-knockout mice [13]. Cysteamine also causes the regression of existing lesions in these mice, decreasing the levels of ceroid and oxidised phospholipids and increasing markers of lesion stability [23]. We do not know if cysteamine protects against atherosclerosis in humans, but there is a striking inverse relationship between arterial calcification and the duration of cysteamine therapy in cystinosis patients [24].

The effects of the thiol cysteine and the disulfide cystine on LDL oxidation are complex. They have been shown sometimes to have antioxidant activity and delay the formation of conjugated dienes in LDL incubated with copper [25,26,27,28] and cysteine decreases the generation of thiobarbituric acid-reactive substances (TBARSs) (mainly malondialdehyde) when LDL is incubated with macrophages in Ham’s F10 medium [25] or copper in Hanks’ balanced salt solution [29]. Cysteine and cystine greatly inhibit the binding of copper to LDL in a 3-(N-morpholino)propanesulfonic acid (MOPS) buffer of pH 7.4 [26].

The thiol cysteine and the disulfide cystine can also have pro-oxidant, as well as antioxidant, effects. LDL oxidation in Ham’s F10 medium is increased by cysteine in terms of TBARS, electrophoretic mobility of LDL and macrophage uptake [30,31]. Cysteine increases hydroperoxides, TBARS and macrophage uptake of LDL incubated with iron in Hanks’ balanced salt solution at acidic pH [29]. Cysteine can be an antioxidant for unoxidized LDL but a pro-oxidant for partially oxidised LDL [26]. LDL oxidation by cells is dependent on cystine, which might be reduced by the cells to cysteine or other thiols, which might be released by the cells and generate superoxide in the extracellular space [32,33] or reduce transition metal ions [34] and form thiyl radicals [31].

**Figure 1:**
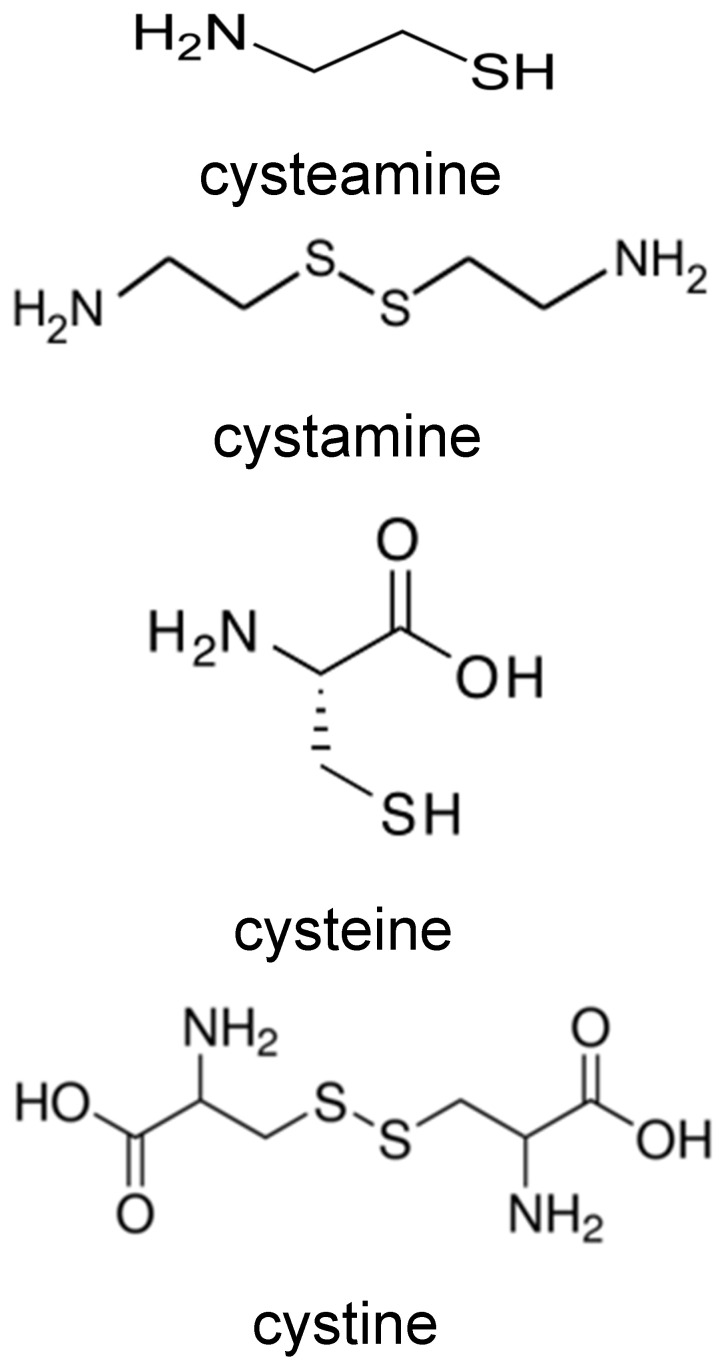
Structures of cysteamine, cystamine, cysteine and cystine.

Lysosomes contain a transporter for the thiol cysteine (MFSD12) [35] and the disulfide cystine (cystinosin) [36] and are known to contain cysteine [37]. Cysteine and cystine would also be produced by the proteolysis of proteins in lysosomes. As lysosomes contain redox-active iron [18] and possibly also contain copper [19], we have investigated the effects of cysteamine, its oxidation product cystamine, together with their analogues cysteine and cystine (those structures are shown in Figure 1) on LDL oxidation by iron or copper at lysosomal pH and pH 7.4.

## 2. Materials and Methods

### 2.1. Materials

Cysteamine hydrochloride, cysteine hydrochloride monohydrate, cystamine dihydrochloride and cystine dihydrochloride were obtained from Sigma-Aldrich, Gillingham, UK. Other chemicals and reagents were purchased from Sigma-Aldrich or Fisher Scientific Ltd., Loughborough, UK.

### 2.2. LDL Isolation

LDL was isolated from blood taken from healthy volunteers after overnight fasting using EDTA (final concentration 3 mmol/L) as the anticoagulant. LDL (1.019 to 1.063 g/mL) was isolated from plasma by sequential density ultracentrifugation at 4 °C, as described in detail previously [38]. Briefly, the density of plasma was adjusted to 1.019 g/mL by adding KBr and dialysing against a solution of exactly this density, and the VLDL and IDL were removed by floatation in an ultracentrifuge. The plasma was then adjusted to a density of 1.063 g/mL in a similar way, and the LDL floated to the top of the tubes by ultracentrifugation. The LDL was washed to remove albumin by adjusting the density again to 1.063 g/mL by dialysis and ultracentrifugation. LDL was stored in the dark at 4 °C and used within 1 month. We obtained permission to take blood from healthy volunteers for LDL isolation. The study was conducted in accordance with the Declaration of Helsinki and approved by the Research Ethics Committee of the University of Reading (Project No. 12/07) on 2 February 2012. Informed consent was obtained from all subjects involved in the study.

### 2.3. Measuring Oxidation of LDL by Spectrophotometry at 234 nm

The quartz cuvettes used to measure oxidation in the spectrophotometer were cleaned thoroughly to ensure any contaminating iron or copper was removed, as described in detail previously [39]. Briefly, the cuvettes were washed with detergent, rinsed in water and then ethanol and more water, incubated with the strong iron and copper chelator diethylenetriaminepentaacetic acid, rinsed with ultrapure water and then ethanol to dry.

The formation of conjugated dienes during LDL oxidation was measured every minute overnight at 37 °C using UV at 234 nm [40,41] in a Cary 60 (Agilent, Stockport, UK) or Lambda Bio 40 (PerkinElmer, High Wycombe, UK) spectrophotometer. LDL was diluted to 50 μg protein/mL in 150 mM NaCl/10 mM sodium acetate, pH 4.5 or 150 mM NaCl/10 mM MOPS buffer, pH 7.4 and oxidised using freshly dissolved 5 µM FeSO_4_ or 5 μM CuSO_4_. The buffers had previously been stirred overnight at 4 °C with washed Chelex-100 (0.1 g/L) to remove contaminating transition metals and the Chelex was removed using a paper filter. The attenuance at time zero was subtracted from all the later values. Where needed, the attenuances of cuvettes incubated with all components except LDL were subtracted from the appropriate attenuances in the presence of LDL.

### 2.4. Statistical Analysis

Data are presented as the mean ± SEM of at least 3 independent experiments. Comparisons were made using a one-way ANOVA test followed by a Dunnett’s post hoc test. Differences were considered significant at *p* < 0.05.

## 3. Results

### 3.1. Effects of Buffer on LDL Oxidation by Iron at pH 4.5

**Figure 2:**
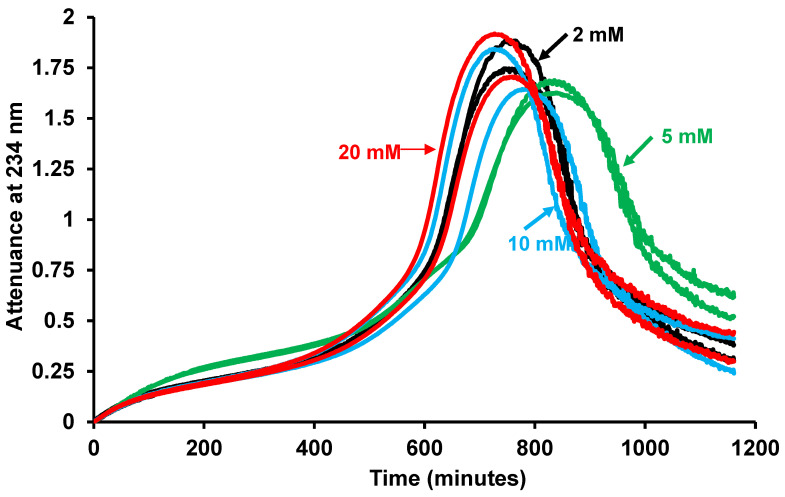
Lack of effect of acetate concentration on oxidation of LDL by iron at pH 4.5. LDL (50 µg protein/mL) was incubated with 5 µM FeSO_4_ at 37 °C in 150 mM NaCl containing 2 mM (black), 5 mM (green), 10 mM (blue) or 20 mM (red) sodium acetate buffer (pH 4.5) and oxidation was monitored by measuring the change in attenuance at 234 nm against appropriate reference cuvettes. A representative experiment from three independent experiments is shown. The results were compared by a one-way ANOVA test, and there was no significant effect of acetate concentration on the time taken to reach an attenuance of 0.4.

LDL oxidation by 5 µM FeSO_4_ was measured in a buffer consisting of 150 mM NaCl/10 mM sodium acetate. As there is a 2000 molar excess of acetate ions, many of which will have a negative charge, to the positively charged iron ions, we wondered if there was any interaction between these two ions that might affect the ability of iron to oxidise LDL. We therefore altered the concentration of acetate in the buffer (keeping the pH constant at 4.5) to see if this would affect the rate of LDL oxidation (Figure 2). As described before [42], there was sometimes a short lag phase, a rapid oxidation phase, a slow oxidation phase, an aggregation phase (in which LDL aggregates are formed and scatter the beam of UV in the spectrophotometer) and a sedimentation phase (in which the attenuance decreases due to the LDL aggregates sinking beneath the beam of UV). The term attenuance is used because the beam of UV is attenuated both by absorbance by the conjugated dienes formed from polyunsaturated fatty acyl moieties during LDL oxidation and later by scattering of the UV by LDL aggregates. The increase in attenuance up to about 0.8 is mainly due to UV absorbance by conjugated dienes, whereas that above about 0.8 is mainly due to UV scattering [42]. There was no significant effect on the rate of LDL oxidation when the acetate concentration was increased from 2 to 5, 10 or 20 mM, as measured by the time taken to increase the attenuance by 0.4 (Figure 2). We chose to use an attenuance increase of 0.4 because this corresponds to about half the maximum extent of lipid peroxidation.

**Figure 3:**
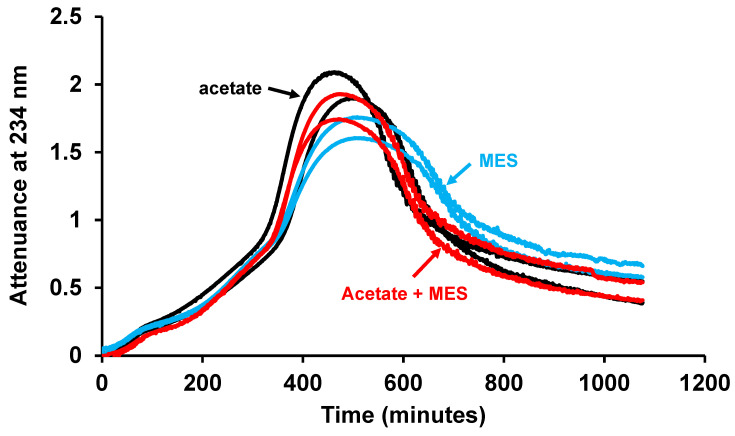
LDL oxidation by iron in acetate and MES buffers of pH 4.5. LDL (50 µg protein/mL) was incubated with 5 µM FeSO_4_ at 37 °C in 150 mM NaCl containing 10 mM sodium acetate (black) or 150 mM NaCl containing 10 mM MES (blue) or 150 mM NaCl containing 10 mM sodium acetate plus 10 mM MES (red), all of pH 4.5. Oxidation was monitored by measuring the change in attenuance at 234 nm against appropriate reference cuvettes. A representative experiment from three independent experiments is shown. The results were compared by a one-way ANOVA test and there was no significant effect of the type of buffer on the time taken to reach an attenuance of 0.4.

There was still a 400-fold excess of acetate over iron with an acetate concentration of 2 mM, so we compared LDL oxidation in buffers of MES (2-(N-morpholino) ethanesulfonic acid; 10 mM), acetate (10 mM), or both acetate (10 mM) and MES (10 mM), all of pH 4.5. There was no significant difference in the rate of LDL oxidation in these three buffers (Figure 3). The addition of 10 mM acetate to the MES buffer did not affect the rate of oxidation of LDL, and thus, acetate did not interact with iron to alter its ability to oxidise LDL.

### 3.2. Effect of Cysteamine, Cysteine, Cystamine and Cystine on LDL Oxidation by Iron at pH 4.5

The peak plasma concentrations of the thiol cysteamine in cystinosis patients treated with this drug are about 70 μM [43]. Lysosomal concentrations of cysteamine would be expected to be much higher, however, as cysteamine is a lysosomotropic compound and the protonation of its amine group at acidic pH would trap it within lysosomes [44]. The free plasma concentrations of the thiol cysteine in humans are about 7 µM, and for the disulfide cystine, it is about 25 µM [45], but the concentrations of cysteine and cystine in lysosomes are unknown. In order to compare the effects of thiols and disulfides directly, we used a concentration of 100 µM for each, as a practical compromise. This concentration would likely be less than the concentration of cysteamine in lysosomes, but might possibly exceed the lysosomal concentrations of the other compounds tested.

**Figure 4:**
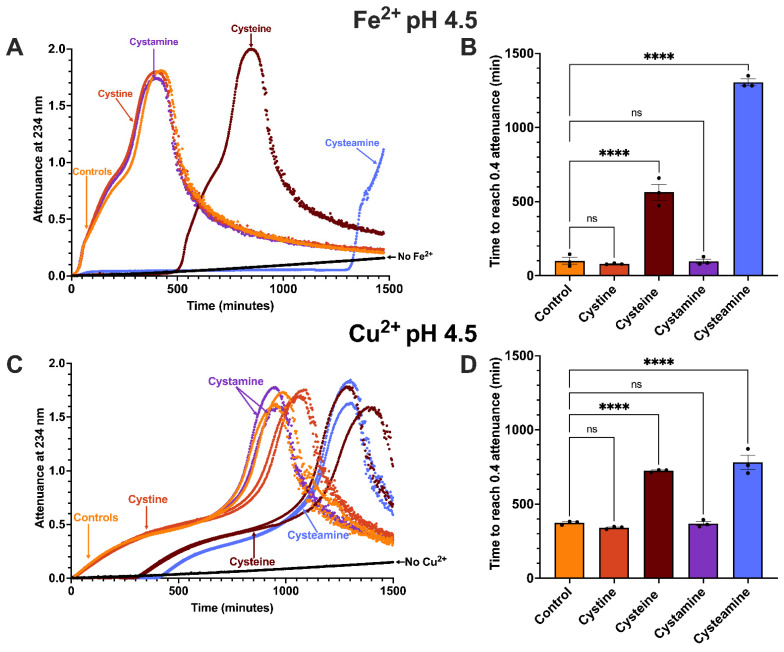
Effect of cysteamine, cysteine, cystamine and cystine on LDL oxidation by iron or copper at pH 4.5. LDL (50 µg LDL protein/mL) was incubated at 37 °C with 5 µM FeSO_4_ (**A**) or 5 µM CuSO_4_ (**C**) at pH 4.5 alone (orange) or in the presence of 100 µM cysteamine (blue), cysteine (brown), cystamine (purple) or cystine (red) or in the absence of FeSO_4_ or CuSO_4_ (black). Oxidation was monitored by measuring the change in attenuance at 234 nm every minute. The times taken for the attenuance to increase by 0.4 are shown for FeSO_4_ (**B**) or CuSO_4_ (**D**) (mean ± SEM of 3 independent experiments) and were compared by a one-way ANOVA test followed by a Dunnett’s post hoc test. ns not significant, **** *p* < 0.0001.

LDL oxidation by ferrous iron at pH 4.5 was greatly inhibited by the thiol cysteamine (100 µM), in agreement with previous work [13,46] (Figure 4). Cysteamine produced a very prolonged lag phase, followed by a similar pattern of oxidation to that observed in the absence of cysteamine. Cysteamine increased the time taken to reach an attenuance of 0.4 by 12.9-fold (*p* < 0.0001). Cysteine has a similar structure to cysteamine but contains a carboxylate group (Figure 1). Cysteine (100 µM) inhibited LDL oxidation by iron at pH 4.5 (*p* < 0.0001), but not for as long as did cysteamine, increasing the time taken to reach an attenuance of 0.4 by 5.6-fold (Figure 4).

Cystamine is the disulfide oxidation product of cysteamine (Figure 1). We have shown previously that LDL oxidation by copper at pH 7.4 is inhibited strongly by both the thiol cysteine and its disulfide oxidation product cystine [26]. We therefore tested whether cystamine and cystine could inhibit the oxidation of LDL by iron at pH 4.5. Surprisingly, LDL oxidation was not affected by either cystamine or cystine (100 μM) at pH 4.5 (Figure 4).

### 3.3. Effect of Cysteamine, Cysteine, Cystamine and Cystine on LDL Oxidation by Copper at pH 4.5

LDL oxidation by copper at pH 4.5 gave a similar profile to that given by iron at the same pH, which is oxidation, followed by aggregation and sedimentation (Figure 4). The oxidation of LDL was delayed to a similar extent by 100 µM cysteamine (2.09-fold; *p* < 0.0001) and cysteine (1.94-fold; *p* < 0.0001), both thiols, unlike LDL oxidation by iron at pH 4.5 in which LDL oxidation was delayed more by cysteamine than by cysteine (Figure 4).

The disulfides cystamine and cystine did not have much effect on LDL oxidation by copper at pH 4.5, as was the case with iron at pH 4.5 (Figure 4).

### 3.4. Effect of Cysteamine, Cysteine, Cystamine and Cystine on LDL Oxidation by Iron at pH 7.4

**Figure 5:**
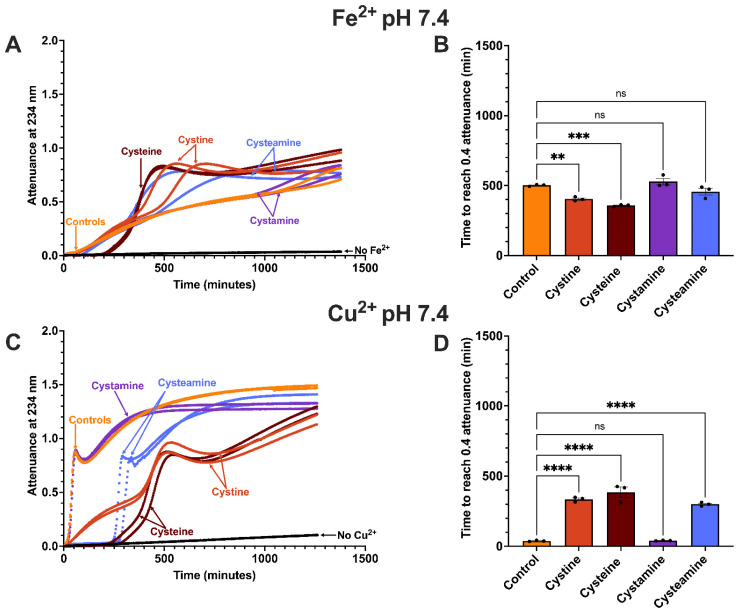
Effect of cysteamine, cysteine, cystamine and cystine on LDL oxidation by iron or copper at pH 7.4. LDL (50 µg LDL protein/mL) was incubated at 37 °C with 5 µM FeSO_4_ (**A**) or 5 µM CuSO_4_ (**C**) at pH 7.4 alone (orange) or in the presence of 100 µM cysteamine (blue), cysteine (brown), cystamine (purple) or cystine (red) or in the absence of FeSO_4_ (black). Oxidation was monitored by measuring the change in attenuance at 234 nm every minute. The times taken for the attenuance to increase by 0.4 are shown for FeSO_4_ (**B**) or CuSO_4_ (**D**) (mean ± SEM of 3 independent experiments) and were compared by a one-way ANOVA test followed by a Dunnett’s post hoc test. ns not significant, ** *p* < 0.01, *** *p* < 0.001, **** *p* < 0.0001.

Ferrous iron can oxidise LDL effectively at pH 7.4 in a MOPS buffer (Figure 5) but not in a buffer containing a high concentration of phosphate [42]. Oxidised LDL does not aggregate at pH 7.4, in contrast to pH 4.5, probably because it has a strong net negative charge repelling the LDL particles [42]. The thiol cysteamine (100 µM) did not inhibit LDL oxidation by ferrous iron at pH 7.4 and actually increased it after about 300 min. Another thiol cysteine (100 µM) strongly inhibited LDL oxidation for about 200 min and then switched to being a pro-oxidant, so that the extent of LDL oxidation was greater in the presence of cysteine at the later time points. The disulfide cystamine did not affect LDL oxidation. Cystine, another disulfide, also had no effect on LDL oxidation initially, but increased the rate of LDL oxidation after about 400 min.

### 3.5. Effect of Cysteamine, Cysteine, Cystamine and Cystine on LDL Oxidation by Copper at pH 7.4

LDL oxidation by copper at pH 7.4 gave the expected and very well-known oxidation profile consisting of a rapid oxidation phase, followed by a decomposition phase and then a slow oxidation phase due to the oxidation of the sterol nucleus in cholesterol and cholesteryl esters (Figure 5) [40,47]. The thiol cysteamine (100 µM) gave a relatively long delay before the oxidation of LDL commenced and the oxidation then followed a similar profile and rate as in the absence of cysteamine. The time to reach an attenuance of 0.4 was increased by 7.8-fold by cysteamine (*p* < 0.0001). The corresponding fold increase at pH 4.5 was 2.1, indicating that cysteamine was a more effective antioxidant at pH 7.4. The thiol cysteine (100 µM) delayed the oxidation of LDL by copper at pH 7.4 by 10.1-fold (*p* < 0.0001), similar to that given by cysteamine, but continued to partially inhibit the oxidation after the phase of total oxidation ended, in contrast to the effects of cysteamine.

The disulfide cystamine (100 µM) did not affect the oxidation of LDL by copper at pH 7.4, in contrast to another disulfide cystine, which inhibited the oxidation, although the inhibition was only partial (*p* < 0.0001) (Figure 5).

## 4. Discussion

LDL can be oxidised by iron and possibly by copper in the lysosomes of macrophages [11]. Lysosomes in foam cells in human atherosclerotic lesions contain catalytically active iron [15,16,17,18], and lysosomes in other cells are known to contain copper [19], but their concentrations are unknown and might vary depending on the contents of the lysosomes and their pH, as this might affect the delivery of these metals to them by autophagy or endocytosis.

To study the mechanisms of LDL oxidation and the effects of various oxidants and antioxidants on this oxidation, we incubated LDL with iron in a NaCl/sodium acetate buffer of pH 4.5 (about lysosomal pH) in a spectrophotometer and measured the formation of conjugated dienes at 234 nm [11,42]. We show here that this buffer does not interfere with the oxidation of LDL. Firstly, increasing the concentration of acetate from 2 to 20 mM did not affect the rate of LDL oxidation (Figure 2). Secondly, the rate of LDL oxidation by iron in an acetate buffer was similar to that in a MES buffer and adding acetate to the MES buffer did not affect the rate of oxidation (Figure 3).

We compared the inhibition by cysteamine and related compounds of LDL oxidation at lysosomal pH and pH 7.4, the pH of plasma and normal interstitial fluid. The thiols cysteamine and cysteine totally inhibited the oxidation of LDL by ferrous iron at pH 4.5 for a long time (Figure 4). This might have been due to these compounds scavenging superoxide radicals (O_2_^•−^), hydroperoxyl radicals (HO_2_•) or radicals generated in LDL. The enthalpy changes for the molecular stationary structures involved in the oxidation of cysteine by the superoxide radical range from −152 to −59 kJ/mol [48].

Fe^2+^ + O_2_ → Fe^3+^ + O_2_^•−^

O_2_^•−^ + H^+^ ↔ HO_2_• (pK_a_ 4.8)

^+^NH_3_CH_2_CH_2_S^−^+ X• + H^+^ ↔ ^+^NH_3_CH_2_CH_2_S• + XH

The hydroperoxyl radical formed at acidic pH is a much more reactive radical than the superoxide radical [46,49] and is not charged, unlike the superoxide radical, and therefore has access to the polyunsaturated lipids of LDL.

After the inhibitory phase, the oxidation started at a similar rate to that in the absence of an added thiol (Figure 4). This was presumably because the thiol group had been consumed and the oxidation could then proceed. The cysteamine radical reacts very rapidly with ionised cysteamine to form the cystamine disulfide radical anion [50].

^+^NH_3_CH_2_CH_2_S• + ^+^NH_3_CH_2_CH_2_S^−^↔ (^+^NH_3_CH_2_CH_2_SSCH_2_CH_2_NH_3_^+^)•^−^

This radical can reduce molecular oxygen to form the disulfide cystamine and the superoxide radical.

(^+^NH_3_CH_2_CH_2_SSCH_2_CH_2_NH_3_^+^)•^−^ + O_2_ ↔ ^+^NH_3_CH_2_CH_2_SSCH_2_CH_2_NH_3_^+^ + O_2_^•−^

The duration of inhibition was greater for cysteamine than for cysteine, implying that cysteamine was consumed more slowly than cysteine. This might possibly be because cysteamine is consumed more slowly than is cysteine by reactions not directly related to the inhibition of LDL oxidation. The reason for this is unknown, but it might be relevant that cysteine, unlike cysteamine, contains a carboxylate group (Figure 1) and that the pK_a_ values of their thiol groups (the pH at which the thiol group is 50% in its negatively charged form) differ, being 8.3 for cysteamine [51] and 8.6 for cysteine [52].

^+^NH_3_CH_2_CH_2_SH ↔ ^+^NH_3_CH_2_CH_2_S^−^+ H^+^ pK_a_ 8.3

^+^NH_3_CH_2_(COO^−^)CH_2_SH ↔ ^+^NH_3_CH_2_(COO^−^)CH_2_S^−^+ H^+^ pK_a_ 8.6

The thiol group of cysteamine would be 11.2% ionised at pH 7.4 and 0.016% ionised at pH 4.5, according to the Henderson-Hasselbalch equation. The thiol group of cysteine would be 5.9% ionised at pH 7.4 and 0.0079% ionised at pH 4.5.

The disulfides cysteamine and cystine did not affect LDL oxidation by iron at pH 4.5 (Figure 4), presumably because they do not contain a thiol group.

The thiols cysteamine and cysteine inhibited LDL oxidation by copper at pH 4.5 for prolonged and similar times, whereas cysteamine inhibited for longer than cysteine with iron at pH 4.5 (Figure 4). After the inhibition phase with copper at pH 4.5, the oxidation of LDL started and had a similar rate and kinetics to that in the absence of cysteamine or cysteine (Figure 4).

The disulfides cystamine and cystine had little effect on the oxidation of LDL by copper at pH 4.5 (Figure 4).

In marked contrast to its effect at pH 4.5, the thiol cysteamine (100 µM) did not inhibit LDL oxidation by ferrous iron at pH 7.4 and even increased the rate of LDL oxidation later in the incubation (Figure 5). This is surprising because antioxidants for LDL are usually more effective at pH 7.4 than at acidic pH [39,46]. Iron would recycle between the ferrous and ferric states during LDL oxidation and the pro-oxidant effect of cysteamine later in the incubation period might have been due to cysteamine reducing ferric iron back to ferrous iron before all the cysteamine was consumed.

^+^NH_3_CH_2_CH_2_S^−^+ Fe^3+^ → ^+^NH_3_CH_2_CH_2_S• + Fe^2+^

LDL oxidation by iron at pH 4.5 is driven by ferrous iron, rather than by ferric iron [42].

The thiol cysteine inhibited the oxidation of LDL by iron at pH 7.4 completely at first and then became pro-oxidant so that the extent of LDL oxidation later in the incubation exceeded that of LDL incubated with iron alone (Figure 5). The initial inhibition might have been due to cysteine scavenging superoxide radicals or radicals generated in the LDL and the pro-oxidant effect might have been due to cysteine reducing ferric iron back into ferrous iron.

The disulfide cystamine had no effect on LDL oxidation by iron at pH 7.4, as was the case at pH 4.5.

Copper, in contrast to iron, oxidises LDL much faster at pH 7.4 than pH 4.5 (Figure 5). The thiol cysteamine inhibited the oxidation of LDL by copper for a long time at both pH values completely, but the fold inhibition was greater at pH 7.4 (Figure 4 and Figure 5). The decreased fold inhibition of LDL oxidation by cysteamine at acidic pH might possibly be explained by the decreased ionisation of the thiol group of cysteamine at acidic pH if the ionised form of the thiol (-S^−^) were to be a better antioxidant than the nonionised form (-SH) [53].

After the total inhibition phase caused by cysteamine, the oxidation of LDL took place at a similar rate to that seen in the absence of cysteamine at pH 7.4, as was the case at pH 4.5. Cysteine also inhibited LDL oxidation completely at pH 7.4 for a long time, in agreement with previous studies [25,26], but once the oxidation commenced, its rate was less than that seen in the absence of a thiol.

The disulfide cystine partially inhibited the oxidation of LDL throughout the entire incubation. This might explain why cysteine decreased the rate of LDL oxidation later in the incubation, as cysteine would have been converted to cystine when it acted as an antioxidant. It might also explain why the inhibition by cysteine later in the incubation was less than the inhibition by cystine early in the incubation, as two molecules of cysteine would have been converted to only one molecule of cystine. Cysteine and cystine might have inhibited LDL oxidation by binding copper, as we have shown previously that at 100 µM they both decreased the binding of copper from 32 atoms per LDL particle to 1 atom per particle [26]. The carboxylate group of cystine might be involved in binding copper, as cystamine, which does not contain a carboxylate group, did not inhibit LDL oxidation by copper at pH 7.4.

## 5. Conclusions

In summary, we have shown that the thiol cysteamine inhibited LDL oxidation by ferrous iron at pH 4.5 and by copper at pH 4.5 and 7.4. The thiol cysteine inhibited LDL oxidation by ferrous iron and copper at both pH 4.5 and 7.4. Cysteamine was a more effective antioxidant for LDL oxidation by copper at pH 7.4 than at pH 4.5, but would still be expected to have a large inhibitory effect on LDL oxidation in lysosomes because it accumulates to high concentrations in these organelles due to proton trapping. The disulfide oxidation product of cysteamine, cystamine, did not inhibit LDL oxidation by ferrous iron or copper at either pH, but the disulfide oxidation product of cysteine, cystine, inhibited LDL oxidation by copper at pH 7.4. The lysosomal oxidation of LDL might be important in atherosclerosis [11,13,23]. This in vitro study shows that the effects of thiols and disulfides on LDL oxidation depend on both the metal ion catalysing the oxidation and on the pH to which the LDL is exposed.

## Data Availability

The original contributions presented in this study are included in the article. Further inquiries can be directed to the corresponding author.
